# Influence of Network Structure on the Crystallization Behavior in Chemically Crosslinked Hydrogels

**DOI:** 10.3390/polym10090970

**Published:** 2018-09-01

**Authors:** Zhenfang Zhang, Qian Li, Cigdem Yesildag, Christoph Bartsch, Xiaoyuan Zhang, Wei Liu, Axel Loebus, Zhiqiang Su, Marga C. Lensen

**Affiliations:** 1 State Key Laboratory of Chemical Resource Engineering, Beijing University of Chemical Technology, Beijing 100029, China; lawrencezzf@hotmail.com (Z.Z.); 2017750003@mail.buct.edu.cn (Q.L.); xiaoyuangem@163.com (X.Z.); viviwliu@163.com (W.L.); 2 Beijing Key Laboratory of Advanced Functional Polymer Composites, Beijing University of Chemical Technology, Beijing 100029, China; 3 Technische Universität Berlin, Institut für Chemie, Nanostrukturierte Biomaterialien, Sekr. TC 1, Straße des 17. Juni 124, 10623 Berlin, Germany; cigdem.yesildag@tu-berlin.de (C.Y.); corynebacter@hotmail.de (C.B.); aloebus@hotmail.com (A.L.)

**Keywords:** crystallization, PEG-based hydrogels, network structure, step-growth polymerization

## Abstract

The network structure of hydrogels is a vital factor to determine their physical properties. Two network structures within hydrogels based on eight-arm star-shaped poly(ethylene glycol)(8PEG) have been obtained; the distinction between the two depends on the way in which the macromonomers were crosslinked: either by (i) commonly-used photo-initiated chain-growth polymerization (8PEG–UV), or (ii) Michael addition step-growth polymerization (8PEG–NH_3_). The crystallization of hydrogels is facilitated by a solvent drying process to obtain a thin hydrogel film. Polarized optical microscopy (POM) results reveal that, while in the 8PEG–UV hydrogels only nano-scaled crystallites are apparent, the 8PEG–NH_3_ hydrogels exhibit an assembly of giant crystalline domains with spherulite sizes ranging from 100 to 400 µm. Scanning electron microscopy (SEM) and atomic force microscopy (AFM) analyses further confirm these results. A model has been proposed to elucidate the correlations between the polymer network structures and the crystallization behavior of PEG-based hydrogels.

## 1. Introduction

Synthetic hydrogels, i.e., cross-linked polymer networks that are insoluble in water yet capable of imbibing water have been widely used as biomaterials in a diverse range of applications [1,2,3,4,5,6,7], such as drug delivery, biosensors, tissue engineering, and wound healing, owing to their excellent biocompatibility, tunable chemical and physical properties, and capability of incorporating bioactive molecules. With the similarity to biological soft tissue, hydrogels, which are also soft and hydrated, could be an ideal substitute for biological soft tissue. Nevertheless, synthetic hydrogels generally have poor functionality. This inherent drawback is primarily due to biological tissue having a sophisticated and multifunctional structure, whereas the network structures of most synthetic hydrogels are simple and inhomogeneous in their spatial distribution of crosslinks, and exhibit topological defects [8,9].

Among various types of polymeric building blocks for hydrogel synthesis, poly(ethylene glycol) (PEG) is one of the most popular polymers, due to the fact that it is hydrophilic, non-toxic, biologically inert, and electrically neutral [10,11]. PEG has been applied in a wealth of fields, such as biomaterials and energetic materials [12,13,14,15,16], as well as numerous industrial applications [17,18,19,20]. It is well-known that a strong link is present between material functions and physical properties, such as crystallinity and network structures. So far, the crystallization and melting behavior of the PEG homopolymer has been extensively studied [21,22,23,24,25,26,27]. Nevertheless, not much research has focused on the crystallization behavior of PEG-based hydrogels. Although the long ordered structure has been found in liquid crystalline physical hydrogels [28,29,30], the crystallinity of chemically-crosslinked PEG-based hydrogels has not been reported until now, probably due to the heterogeneous nanoscale structure of hydrogels fabricated via commonly-used chain growth polymerization, which only leads to nanoscale crystal structure formation [31,32].

Because of the numerous applications of PEG-based hydrogels, understanding the correlation between the crystallization behavior and the network structure of PEG-based hydrogels is of both fundamental and commercial importance. Very recently, a new class of eight-arm star-shaped poly(ethylene glycol)-based hydrogels (8PEG–NH_3_) formed by an amine Michael-type addition reaction between unsaturated carbon-carbon double bonds (acrylate end groups) and reactive amine species has been developed in our lab. By variation of the crosslinking parameters, the degree of residual functional groups, the swelling degree, and the mechanical properties of the resulting gels can be elegantly tuned [33,34]. In the present study, the crystallization properties of these novel hydrogels have been examined by polarized optical microscopy (POM), differential scanning calorimetry (DSC), wide-angle X-ray diffraction (WAXD), scanning electron microscopy (SEM), as well as atomic force microscopy (AFM). For comparison, 8PEG hydrogels that were synthesized by photoinitiated radical crosslinking (UV-curing) were analyzed, revealing remarkable differences. The reasons for the different morphologies will be discussed in terms of the characteristically different polymer network structures.

## 2. Materials and Methods

### 2.1. Materials

All chemicals were purchased from Aldrich (Munich, Germany) and used as received unless stated otherwise. Solvents were at least analytical grade quality. 8arm poly(ethylene glycol) (8PEG–OH, *M*w 15 KDa) was purchased from Jenkem technology USA (Plano, TX, USA). 8-arm poly(ethylene glycol) acrylate (8PEG) was prepared via the same procedure as we have reported before [33].

### 2.2. Synthesis of 8-arm PEG-Acrylate (8PEG)

First, 8-arm, star-shaped PEG with OH–end groups (8PEG–OH; 15 kDa) and K_2_CO_3_ were dried in a vacuum oven at 100 °C for 4 h. Then, 8PEG–OH (5 g) and K_2_CO_3_ (3 g) were added in 50 mL CH_2_Cl_2_ (DCM) under N_2_-atmosphere. Acryloyl Chloride (1 mL) was added dropwise to the reaction mixture in a water-ice bath. The mixture was stirred at 60 °C for 4 days. The solution was filtered, and then poured into cold petroleum ether (cooled by water-ice). The solution was stirred for 10 min, and then separated to get the crude product. The crude product was dissolved in 50 mL of DCM and then extracted with a saturated NaCl-solution 3 times. The organic layer was collected. The solution was dried by magnesium-sulfate overnight, then filtered to remove MgSO_4_, and subsequently, the solvent was removed under reduced pressure to get the final product as a white solid. Isolated yield (72%). ^1^H-NMR (400 MHz, CDCl_3_): OCH_2_CH_2_O 3.64 ppm (1496H), (C=O)OCH_2_ 4.31 ppm (16H), =C–H trans 5.83ppm (8H), CH=C 6.15 ppm (8H), =C–H cis 6.42 ppm (8H).

### 2.3. Methods

Differential scanning calorimetric (DSC) analyses were employed with a Mettler-Toledo DSC 822e (Mettler-Toledo GmbH, Zurich, Switzerland). Samples with 3–5 mg in weight were encapsulated in aluminum pans. The calibration was performed with indium and hexatriacontane. An ultra-pure nitrogen atmosphere was employed as circulating atmosphere for all tests. The measurement was performed at the heating rate 10 °C/min.

Wide-angle X-ray diffraction (WAXD) analyses were carried out using a D/Max 2500 XB2+/PC X-ray diffractometer (Rigaku, Tokyo, Japan). The scanning angle 2θ was from 10° to 60°. Annealed samples having 50 μm thickness were prepared for WAXD measurements, and all the experiments were carried out at room temperature (25 °C). The casting films on sheet glasses were fixed onto the equipment. The data were collected in every 0.02 s.

Raman Spectroscopy (LABRAM, HR Horiba Scientific, Bensheim, Germany) was conducted on films dried at ambient conditions with an excitation wavelength of 514 nm. Spectra were recorded between 500 and 3000 cm^−1^.

Polarization microscope morphology (POM) observations of the hydrogels were performed with an Olympus optical microscope (Olympus, BX51, Tokyo, Japan), equipped with a Canon EOS40D camera system. The whole processes were carried out in a nitrogen atmosphere.

Scanning electron microscopy (SEM) were taken with a Hitachi S-520 (Hitachi, Tokyo, Japan) using an acceleration voltage of 20 kV and a working distance of 10 mm. The samples were sputtered with gold using a sputter coater (SCD 030, Balzers, Liechtenstein). Pictures were taken using the Digital Image Processing System (2.6.20.1, Point Electronic, Halle, Germany).

An Atomic Force Microscope (AFM, JPK instruments, Nanowizard II, Berlin, Germany) was used in order to measure the topography and surface elasticity of samples in dry and swollen state. Imaging was done in intermittent contact and contact mode using silicon nitride cantilevers (PNP TR, *k* ≈ 0.08 N/m, *f*_0_ ≈ 17 kHz; Nanoworld Innovative technologies) with a chromium-gold coating. Images were edited with NanoWizard IP Version 3.3a (JPK instruments, Berlin, Germany). Samples measured in swollen state were immersed in deionized water prior to measuring.

### 2.4. 8PEG-UV Hydrogel Samples Preparation

Aqueous 8PEG (50 wt %) containing 1% of photinitiator (PI) (1 wt % with respect to the amount of the precursor) were prepared. Subsequently, 50 μL of the 8PEG precursor mixtures were dispensed on a clean glass slide, capped with a cover glass (18 mm × 18 mm; Carl Roth GmbH & Co. KG, Karlsruhe, Germany) and exposed to UV light (λ = 365 nm, Vilber Lourmat GmbH, Eberhardzell, Germany) for 15 min using a working distance of 10 cm, in a nitrogen-filled glovebox. The cured transparent gels were peeled off with tweezers. And then the samples were put on a new and clean glass slide. Finally the water was evaporated and the gels were further dried until constant weight.

### 2.5. 8PEG–NH_3_ Hydrogel Samples Preparation

Aqueous 8PEG (40 wt %) containing 1% of PI (1 wt % with respect to the amount of the precursor) were prepared. 15 μL of ammonium solution (30% NH_3_ in H_2_O) were added to 100 μL of the 8PEG precursor mixtures at room-temperature under vigorous magnetic stirring, and the reaction was allowed to run until the solution turned into a viscous liquid. The resulting liquids were deposited on the clean glass slide, capped with a cover glass (18 mm × 18 mm; Carl Roth GmbH & Co. KG, Karlsruhe, Germany). After 0.5 h, the 8PEG–NH_3_ hydrogel were formed. The cured transparent gels were peeled off with tweezers. And then the samples were put on a new and clean glass slide. Finally the water was evaporated and the gels were further dried until constant weight.

### 2.6. 8PEG Macromonomer Film Preparation

Aqueous 8PEG (50 wt %) containing 1% of PI (1 wt % with respect to the amount of the precursor) were prepared. The solution was stirred at room temperature over 1 h, then casted on a new and clean glass slide. Finally the water was evaporated and the gels were further dried until a constant weight was observed.

## 3. Results and Discussion

### 3.1. Hydrogel Formation and Network Structure

**Figure 1:**
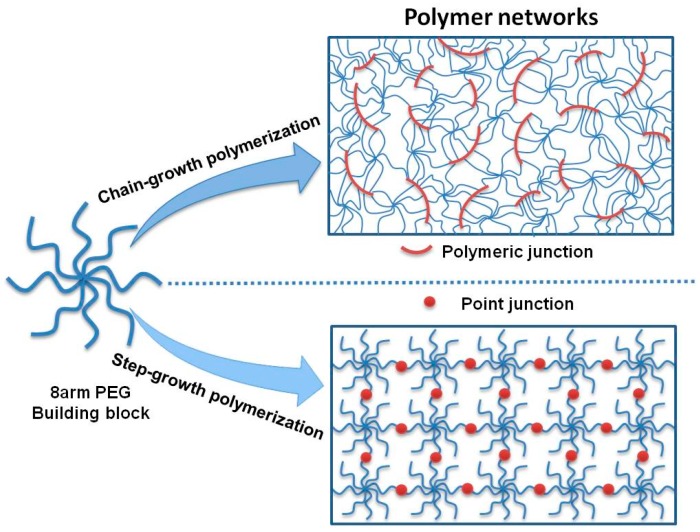
Schematic depiction of 8-arm polymer (as building block) networks structures obtained via chain-growth polymerization resulting in rigid polymeric junctions (**top**) and step-growth polymerization resulting in point junctions (**bottom**).

Herein, two distinctly different PEG hydrogel networks were synthesized, using the two types of crosslinking chemistry, as shown in Figure 1 (top). Firstly, UV-cured 8PEG–based hydrogels (8PEG–UV) were synthesized via photoinitiated chain polymerization (chain-growth polymerization) of 8PEG macromonomers with acrylate end groups. The resulting hydrogels of such a chain-growth polymerization exhibit polymeric junctions (i.e., polyacrylate chains), a high degree of an inhomogeneous spatial distribution of crosslinks, as well as a broad distribution of network-strand lengths [35,36].

Secondly, functional 8PEG–NH_3_ hydrogels were obtained by simply mixing the 8PEG acrylate macromonomers with an ammonium hydroxide solution, which is a Michael-type addition between the acrylate and amine groups, as schematically depicted in the Supplementary Material (Figure S1a). Raman spectroscopy was applied to analyze the amount of residual acrylate end-groups on the gel. The spectrum in in Figure S1b indicates that after gel formation, most of the reactive acrylate groups in the hydrogel in fact have not been consumed, leaving them are available for further functionalization.

In this case, the cross-linking chemistry can be considered a step-growth polymerization of macromonomers, and the resulting hydrogel network is held together by point junctions instead of polyacrylate chains, as was the case for UV-cured polymer networks. Figure 1 schematically explains those differences in the two different network architectures.

In such networks formed via step-growth polymerization (Figure 1 (bottom)) the flexibility of the PEG-chains is largely preserved and relatively homogeneous nanoscale network structures are established [37,38]. In this study, also the 8PEG macromonomer building blocks without crosslinking were investigated for comparison with the crosslinked hydrogels.

The synthesis and characterization of all relevant 8PEG-macromonomers and 8PEG–gels is described partly in the experimental section and further in the Supplementary Material, e.g., the FT-IR spectra are shown in Figure S2.

### 3.2. Spherulites Observation

**Figure 2:**
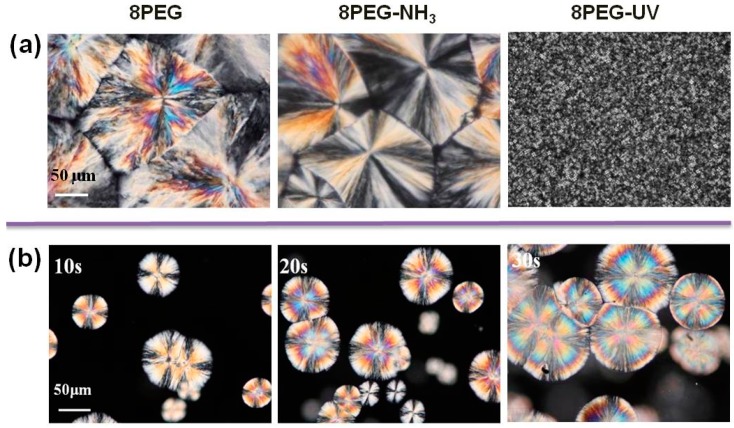
Polarized optical micrographs of (**a**) solid films of 8PEG macromonomer, 8PEG–NH_3_ hydrogel, 8PEG–UV hydrogel; as well as 8PEG–NH_3_ hydrogel (**b**) during the drying process.

As the flexibility of the PEG-chains is expected to play a crucial role in any crystallization process, we made the 8PEG gels solidify (by drying) and studied the internal morphology of the dried polymer films by several analytical tools. Figure 2 depicts the observations made by polarized optical microscopy (POM) for the two different 8PEG hydrogels and the 8PEG macromonomers as a control. Figure 2a reveals significant differences among the three samples; whereas the 8PEG–UV gel shows only tiny structural features, large spherulites are evident for 8PEG–NH_3_ gels, which are quite similar to those seen for the non-crosslinked 8PEG control.

Spherulites are a morphological feature of crystallized polymers and consist of a large number of chain-folded lamellar crystallites, radiating in all directions from a central nucleus with molecular chains oriented tangentially [39,40]. For 8PEG macromonomers (Figure 2a, (left)), the spherulite size of the polymer reached hundreds of microns. Large and well-defined spherulites with sizes of 100–400 μm and the apparent Maltese crosses were also observed for dried films of 8PEG–NH_3_ hydrogel (Figure 2a, (middle)). The similarity of both spherulite patterns indicates that the continuous radial variation of the orientation of the polymer crystal axes is not inhibited by the point junctions in the 8PEG–NH_3_ gels, meanwhile the homogeneous network structures facilitate the polymer chains folding during the crystallization.

In contrast, no large spherulites, but a homogeneous distribution of nano-crystals were observed for the 8PEG–UV hydrogel (Figure 2a, (right)). Such nano-sized crystalline domains could be attributable to the limited flexibility of the polymer chains in those gels formed by chain-growth crosslinking, involving rigid polymeric junctions. Figure 2b shows the growth of spherulites in 8PEG–NH_3_ hydrogel with increasing crystallization time (crystallization temperature is 25 °C), as the water evaporates. At the early stage, spherulites with Maltese crosses appeared in the isotropic (dark) phase, and then the crystallites grew in size and obtained a more closed, spherical shape.

### 3.3. Crystal Structure Characterization

**Figure 3:**
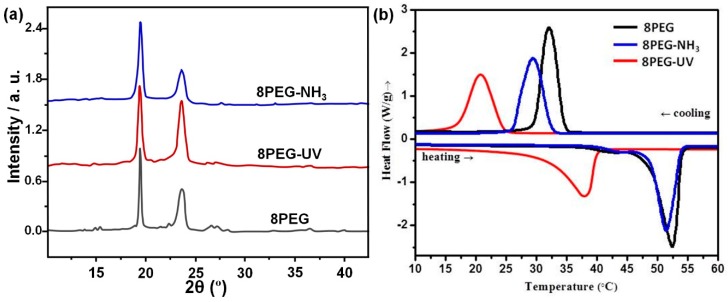
(**a**) WAXD patterns and representative (**b**) DSC curves of 8PEG, 8PEG–NH_3_ and 8PEG–UV.

The crystallization states of 8PEG polymer, 8PEG–UV and 8PEG–NH_3_ hydrogels have been investigated by WAXD. From the patterns in Figure 3a, different diffraction peaks for the PEG crystals can be detected, which correspond to the (120) and (032) planes of PEG crystallites [25], indicating the existence of a monoclinic phase. These diffractions are characteristic to PEG crystals where the PEG chains form a helix structure with 6 Å per cycle [41]. The two typical peaks suggest that PEG chains in all samples are all able to crystallize and form separate crystalline phases. The almost identical diffractograms indicate that no significant difference of the PEG crystalline phases have been found in the hydrogels.

Another method to monitor crystallization and melting behavior is differential scanning calorimetry (DSC). Figure 3b shows the DSC thermographs for the 8PEG polymer and its corresponding hydrogels during heating processes with a heating rate of 10 °C/min. Sharp endothermic peaks are observed and are assigned to the melting temperatures (*T*_m_). Tm of the pure 8PEG polymer is 52.4 ± 0.2 °C (Figure 3b).

It is interesting to note that the melting temperatures of the crosslinked hydrogels are both lower, with the largest deviation being observed for the 8PEG–UV gel. The low values of crystallinity and corresponding melting temperatures can be ascribed to the non-perfect crystal formed in the hydrogels and the irregular polymer chains folding. During the gel formation processes, the PEG chains are kinetically trapped in a confined space considering they are fixed in the gel network. That is why the 8PEG gels exhibit lower crystallinity, and consequently lower melting temperatures. Melting and crystallization temperatures, alongside crystallinity values are listed in Table S1.

Obviously, 8PEG–UV hydrogels show the lowest melting temperature, while the Tm of 8PEG–NH_3_ hydrogels is closest to that of 8PEG polymer. This can be understood by taking into account that in the photocross-linked 8PEG–UV gel matrix most of the PEG chains are cross-linked and confined in the nanoscaled space. In contrast, as shown in Figure S1b, due to incomplete reaction, about 70% of PEG chains in 8PEG–NH_3_ gel matrix are not cross-linked, leaving many residual acrylate end groups on the dangling chains. These unbound chains can be easily folded, which explains the higher degree of crystallinity and correspondingly higher melting temperature. In addition, unlike polyacrylate chains in 8PEG–UV, which form during the chain polymerization process and lead to more amorphous phase, the crosslinking points in 8PEG–NH_3_ (formed according to a step-growth mechanism) hardly have any influence on crystallite growth.

### 3.4. Investigation of the Surface Morphology

**Figure 4:**
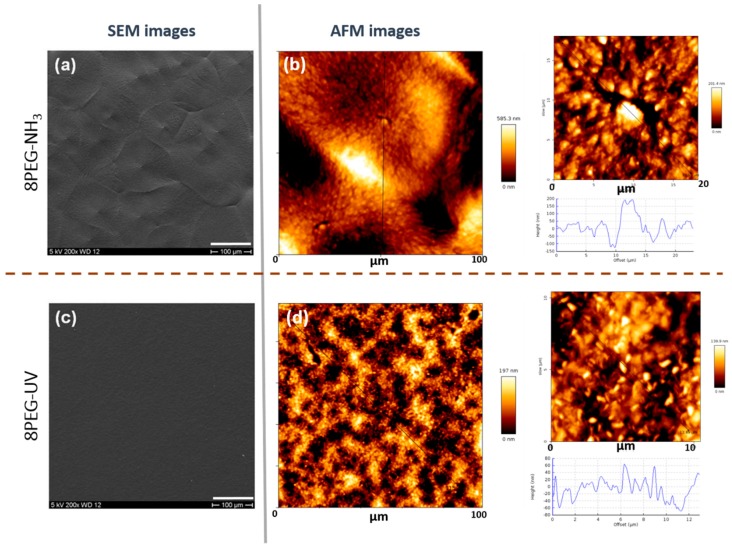
Representative SEM and AFM images of 8PEG–NH3 (**a**,**b**), and 8PEG–UV (**c**,**d**) hydrogels. As-prepared samples were dried in air prior to the measurements (SEM in vacuum and AFM in air). Scale bars (**a**,**c**) 100 µm.

After having analyzed the bulk properties of the three different materials, we were curious to determine if the crystallinity at the nano- or micrometer scale could be detected at the surface of the solidified films. Therefore, we performed surface analyses, i.e., scanning electron microscopy (SEM) and atomic force microscopy (AFM). The results are shown in Figure 4. In Figure 4a, for the 8PEG–NH_3_ hydrogel, the regular spherulites, hundreds of µm in size, with radial stripes that were observed earlier by POM (Figure 2), are indeed visible by SEM. As could be expected on the basis of the POM image of the 8PEG–UV hydrogel, no structural features could be detected on its surface (Figure 4c), implying that the crystals that formed, according to WAXD, must be only nanometer-scaled.

Figure 4 also shows the AFM results of the 8PEG–NH_3_ and 8PEG–UV hydrogels crystallized at room temperature. Figure 4b shows that 8PEG–NH_3_ hydrogel forms regular, large spherulites. In contrast, no regular and highly ordered structures were observed on 8PEG–UV hydrogel surface, as shown in Figure 4d.

Through the close observation from the higher magnification AFM height image and cross-section in Figure 4 (right), irregular, random distributed and nano-sized features were detected. Moreover, the surface topography of 8PEG–NH_3_ was (~1.5 times) larger than that measured for 8PEG–UV, which was probably related to the larger lateral dimensions of the crystals. It should be noted that the AFM imaging was performed on the exposed surfaces of the hydrogel films (i.e., against air), while the POM showed the images of the bulk hydrogels between two glass slides.

Besides letting the as-prepared gels, which contain water from the reaction mixture, dry over the course of several hours in air and measuring the surface morphology by AFM (Figure 4), they were put in a vacuum oven overnight at 40 °C to remove all water, including the hydration mantle from the PEG–gels. AFM studies were carried out to see if this would affect the morphology.

Figure S3 demonstrates that, in this case, there was a rather regular texture observed at the dehydrated surface of the 8PEG–UV gels, whereas the surface of the dehydrated 8PEG–NH3 was amorphous and irregular. Moreover, while the 8PEG–UV surface exhibited a smooth topography (roughness of ~6–10 nm), there was a significant topographic landscape on the 8PEG–NH_3_ gels with height differences in the µm-range (~0.6–1.8 µm). The irregular surface of the 8PEG–NH_3_ gels makes us suspect that the gels may have lost their integrity during the swelling and/or subsequent drying, which is conceivable regarding the hydrolytic lability of the ester moieties at the cross-linking points.

### 3.5. Hydration and Dehydration Dynamics

The dehydrated gels were immersed in water until the equilibrium water content (EWC) was reached, and then analyzed by AFM, Figure S4, (left). These gels with their EWC were left to dry again in air and analyzed by AFM during drying (measured in air), Figure S4 (middle and right). The tightly cross-linked 8PEG–UV gels were mainly featureless and quite smooth; even in the fully hydrated state, the roughness was below 15 nm. Upon drying, some features seemed to appear which could be attributed to nano-crystallites. The 8PEG–NH_3_ gels, on the other hand, exhibited featureless, “fluffy” surfaces with height differences of 1–2 µm; both in the hydrated state as in the drying gels. We tentatively attributed this lack of structure again to the loss of integrity of the 8PEG–NH_3_ upon prolonged incubation in water (for reaching the EWC), which may lead to degradation of the gels.

Nevertheless, as the useful hydrogel properties for application as biomaterials are best taken advantage of in the swollen state, the swelling and drying (or deswelling) behavior of the 8PEG-gels was investigated in more detail, and the results are summarized in the Supplementary Material (Table S1 and inset graphs in Figure S4).

As we know already from experience in handling the gels, and from these and previous analyses [33], we can conclude that the step-growth mechanism leads to less tightly cross-linked hydrogels that swell more and that are softer than the typical, UV-cured PEG-hydrogels (also in hydrated state). Drying of the hydrogels leads to a densification of the hydrogel network and crystallization, as we have focused on in this paper. The swelling of the 8PEG–NH_3_ gels is accompanied by eventual disintegration of the gels, because the ester moieties connecting the cross-linking points between the macromonomers are not stable against hydrolysis. Thus, after prolonged incubation in water, these gels lose their integrity. This can be overcome by applying another round of cross-linking, e.g., by UV-curing, of the remaining acrylate groups on these functional 8PEG–NH_3_ gels [33].

### 3.6. Proposed Crystallization Mechanism

**Figure 5:**
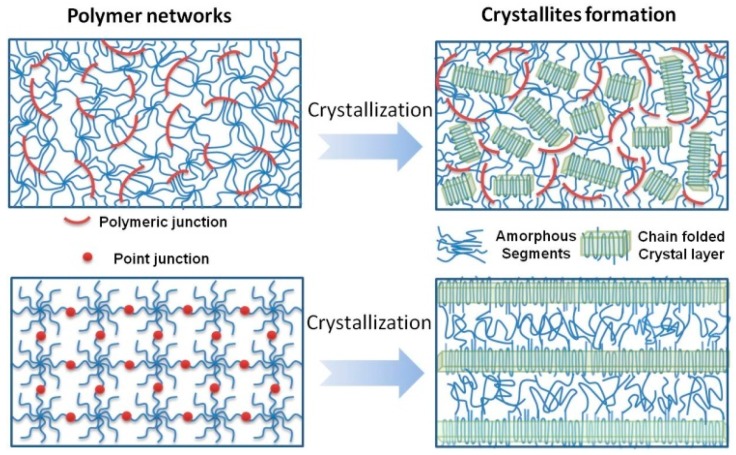
Schematic depiction of the proposed distinct crystallization structures at the supramolecular scale that reside from the two different types of gel network structure.

Considering that the chemical constitution of all three samples, consisting of 8PEG, are the same, the reason for the observed different morphologies in the dried films should be sought in the distinct characteristics of the nanoscopic gel network structures. To clearly explain the difference, we propose a model here to schematically illustrate the formation of different crystalline morphologies in the gel matrices (Figure 5).

Depending on the crosslinking reaction mechanism, Figure 5 illustrates the network structures resulting from the chain-growth polymerization (8PEG–UV) and step-growth polymerization (8PEG–NH_3_) from the same 8PEG precursors. In chain-growth polymerization (8PEG–UV), the propagation of free radicals through multiple carbon-carbon double bonds on the constituting PEG macromonomers results in covalently crosslinked, yet uncontrolled, rigid and polyacrylate chains, corresponding to irregularly distributed cross-linked junctions and consequently network imperfections, such as cycles and entangled chains [35,36]. Clearly, these rigid polyacrylate chains increase the kinetic barrier for polymer chain straightening and disturb the formation of aligned, chain folded lamellae, resulting in reducing the propensity of PEG to form highly ordered crystalline structures. Meanwhile the polymer chains are tightly connected to each other, which confine the crystallization process at the nanoscale, leading to the formation of nanocrystals (Figure 5).

Different from 8PEG–UV gels, the networks of 8PEG–NH_3_ form through a step-growth polymerization of amines and conjugated unsaturated vinyl groups, and two multifunctional monomers with mutually reactive chemical groups are reacted to form one crosslink point, producing fewer structural defects during network formation and more flexible polymer networks, so that the resulting hydrogels possess homogeneous network structures [37,38]. The hydrated and flexible polymer chains and crosslink points are both regularly distributed. When drying, the polymer chains can easily fold and propagate from the center, resulting in the well-defined and long-range ordered spherulites structure in the gel matrix (Figure 5).

## 4. Conclusions

In conclusion, comparison of the crystallization of chemically cross-linked PEG-based hydrogels, either evolved from chain-growth (8PEG–UV) or step-growth (8PEG–NH_3_) polymerization, has revealed significant differences. While WAXD spectra of the 8PEG macromonomer and two 8PEG-based hydrogels did not disclose any significantly different crystalline phases, microscopic observations demonstrated that the sizes of crystalline domains are significantly different. POM, SEM, and AFM studies confirm that the 8PEG–NH_3_ gels exhibit a construction of crystalline domains with sizes of 100–400 µm, whereas in 8PEG–UV the crystalline domains are only nanoscale in size. Thermal investigations by DSC further showed a clear correlation between the degree of crystallinity and the melting temperatures of the two 8PEG hydrogels and the control sample (i.e., 8PEG macromonomer).

All results together consistently support our hypothesis that the network structure of 8PEG hydrogels, which is determined by the crosslinking strategy, greatly influences the scale of crystallites, e.g., large spherulites in a step-growth mechanism or only small nanocrystallites when chain-growth polymerization applies. It is a great insight to understand how the crystallization of chemically crosslinked hydrogels can be controlled at multiple hierarchical levels by (i) the molecular structure, (ii) the crosslinking chemistry, (iii) the supramolecular organization at the nanoscale and (iv) the eventual long-range, microscopic and sub-mm organization into crystal structures.

## Supplementary Materials

The following are available online at http://www.mdpi.com/2073-4360/10/9/970/s1.
