# Supplementary material for: Influence of Network Structure on the Crystallization Behavior in Chemically Crosslinked Hydrogels
Source: Polymers (Basel). 2018 Sep 1;10(9):970. doi: 10.3390/polym10090970 (PMC6403567; doi:10.3390/polym10090970)
Supplement: Supplementary file 1 [file polymers-10-00970-s001.pdf]

polymers

Supporting Information

Influence of Network Structure on the Crystallization Behavior in Chemically Crosslinked Hydrogels

Zhenfang Zhang[1,2,3], Qian Li[1,2], Cigdem Yesildag[3], Christoph Bartsch[3], Xiaoyuan Zhang[1,2], Wei Liu[1,2], Axel Loebus[3], Zhiqiang Su[1,2,*] and Marga C. Lensen[3,*]

1 State Key Laboratory of Chemical Resource Engineering, Beijing University of Chemical Technology, 100029, Beijing, China; suzq@mail.buct.edu.cn (Z.S.)
2 Beijing Key Laboratory of Advanced Functional Polymer Composites, Beijing University of Chemical Technology, 100029 Beijing, China.
3 Technische Universität Berlin, Institut für Chemie, Nanostrukturierte Biomaterialien, Sekr. TC 1, Straße des 17. Juni 124, 10623, Berlin, Germany; Lensen@Chem.TU-Berlin.de (M.C.L)
* Correspondence: suzq@mail.buct.edu.cn (Z.S.); Lensen@Chem.TU-Berlin.de (M.C.L); Tel.: +86- 010-64419060 (Z. S.); +49-30-31429555

1. Materials and Methods

1.1. Materials

All chemicals were purchased from Aldrich and used as received unless stated otherwise. Solvents were at least analytical grade quality. 8arm poly(ethylene glycol) (8PEG-OH, M_w 15KDa) was purchased from Jenkem technology USA.

1.2. Synthesis of 8arm PEG-acrylate (8PEG)

8-arm poly(ethylene glycol) acrylate (8PEG) was prepared via the same procedure as we have reported before [33].

First, 8-arm, star-shaped PEG with OH-end groups (8PEG-OH; 15 kDa) and K_2CO_3 were dried in a vacuum oven at 100 °C for 4 h. Then, 8PEG-OH (5g) and K_2CO_3 (3g) were added in 50 ml CH_2Cl_2 (DCM) under N_2-atmosphere. Acryloyl chloride (1 mL) was added dropwise to the reaction mixture in a water-ice bath. The mixture was stirred at 60 °C for 4 days. The solution was filtered, and then poured into cold petroleum ether (cooled by water-ice). The solution was stirred for 10 min, and then separated to get the crude product. The crude product was dissolved in 50 mL of DCM and then extracted with saturated NaCl-solution for 3 times. The organic layer was collected. The solution was dried by magnesium-sulfate overnight, then filtered to remove $MgSO_4$ and subsequently the solvent was removed under reduced pressure to get the final product as a white solid. Isolated yield (72%). 1^H-NMR (400 MHz, $CDCl_3$): OCH_2CH_2O 3.64 ppm (1496H), (C=O)OCH_2 4.31 ppm (16H), =C-H trans 5.83 ppm (8H), CH=C 6.15 ppm (8H), =C-H cis 6.42 ppm (8H).

1.3. 8PEG-NH3 hydrogel Synthesis

Different amounts of ammonium solution (30% NH_3 in H_2O) were added to the precursor solution of 8-arm poly(ethylene glycol) acrylate (8PEG) with 40% water content at room-temperature under vigorous magnetic stirring until the solution turned into a viscous liquid. Compositions were set in order to receive 12 wt%, 6 wt%, 3 wt%, 1.5 wt% and 0.75 wt% NH_3-8PEG by weight. The resulting liquids were deposited on a glass slide and covered with a glass cover slip. The required time for gelation of 12 wt%, 6 wt%, 3 wt%, 1.5 wt% and 0.75 wt% 8PEG-NH_3 gels are 0.4 h, 0.5 h, 1 h, 2 h and 4 h, respectively.

1.4. UV stabilization of 8PEG-NH3 hydrogel (optional):

For further UV stabilizing the loosely crosslinked and degradable gels, before NH_3 addition, 1% photinitiator (PI) (Irgacure 2959) was added to the reaction mixture. After preparation of 8PEG-NH_3 gels, the gels were exposed to UV light (340 nm) for 15 minutes to obtain stable 8PEG-NH_3-UV gel.

1.5 AFM characterization

AFM measurements were carried out using a Nanowizard II (JPK instruments) in order to measure the topography of samples in dry and swollen state as well as for monitoring the drying process. Imaging was done in air (drying process) and under water (fully swollen samples) in contact mode using a silicon cantilevers (ContAl, $k \approx 0.2$ N/m, $f_0 \approx 13$ kHz; Budget Sensors) with an aluminum reflex coating. Images were edited with NanoWizard IP Version 3.3a (JPK instruments).

2. Materials Characterization

2.1. Raman

The course of the reaction, which follows the Michael-type addition scheme, as depicted in Figure S1a, was monitored in time by rheology. Details can be found in this publication and supporting information: [Zhang, Lensen, Chem. Mater., 2014, 26 (12), pp 3624–3630. DOI: 10.1021/cm500203j].

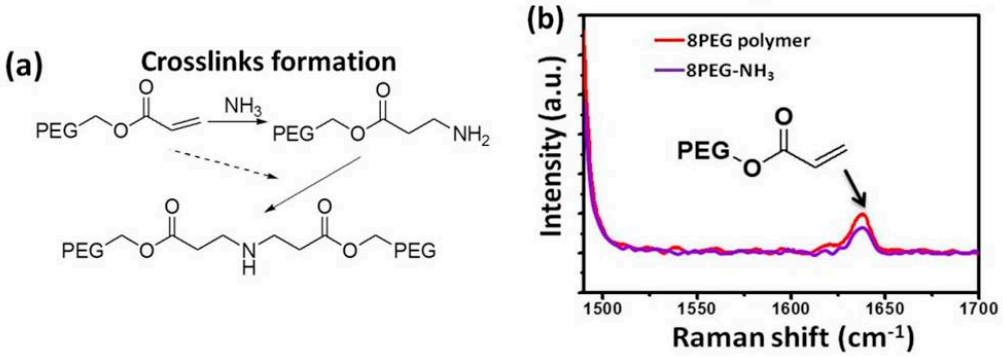

Figure S1. (a) Crosslinks formation through amine-Michael type addition; (b) RAMAN spectra of the residual C=C double bonds of PEG-NH_3 as dangling chains in hydrogel matrix.

Raman spectroscopy was applied to estimate the number of unreacted acrylate groups that typically remained on the gel after reaction with ammonia. Figure S1b depicts the representative spectrum for 3% of NH_3 in this case.

2.2. FT-IR

In addition, FT-IR measurements were performed to analyze the successful substitution of –OH groups for acrylate end-groups, and for the efficiency of the reaction, i.e. the disappearance of acrylate groups in reaction with ammonia. Scheme S1 depicts the molecular structure of the 8PEGacr macromonomers and schematic drawings of the two different gels, while Figure S2 shows the relevant FT-IR spectra.

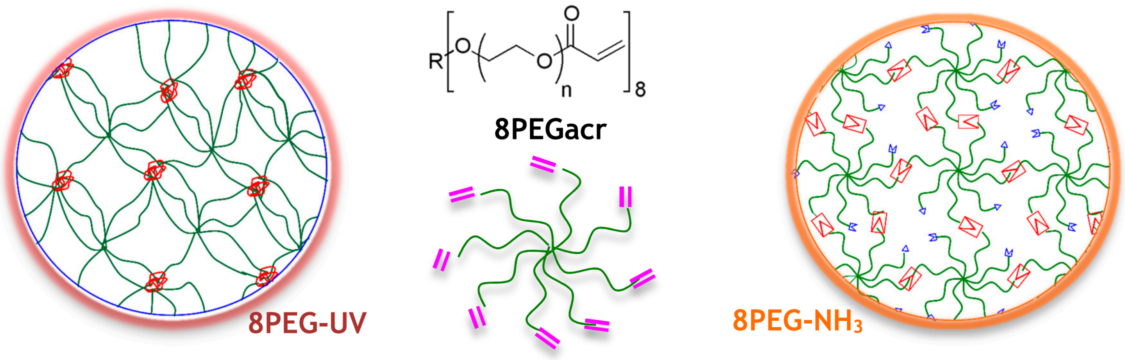

Scheme S1. Cartoon and chemical structure of the 8PEG-macromonomers (n~43) with 8 acrylate end-groups (middle), and schematic representation of the two different network structures in 8PEG-UV and 8PEG-NH_3 gels (left and right, respectively). .

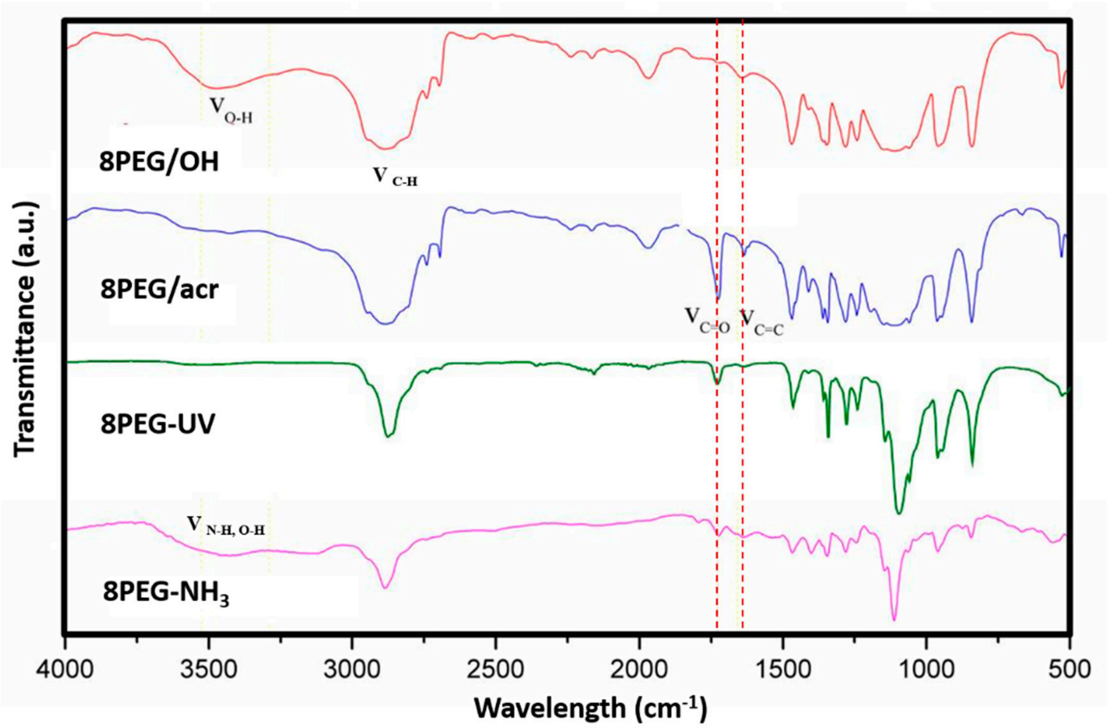

Figure S2 FT-IR spectra of 8PEG-OH and 8PEGacr macromonomers and of 8PEG gels after reaction with ammonia (8PEG-NH3) or by UV-curing (8PEG-UV).

From the spectra in Figure S2, as acrylate groups were modified onto the ends of 8PEG polymers, new peaks of C=O and C=C from acrylate groups, corresponding to 1730 cm^{-1} and 1640 cm^{-1} appeared, but the peak of O-H, corresponding to 3450 cm^{-1} vanished from 8PEG's spectrum, indicated the conversion of end groups from O-H to acrylate had been successfully achieved.

After hydrogel formation, the peaks of C=O were still evident in both spectra of 8PEG-UV and 8PEG-NH_3.

For 8PEG-UV the peak of C=C groups almost disappeared due to the total consumption of C=C groups when UV-curing was conducted, whereas for 8PEG-NH_3, due to the partial consumption of C=C groups with Michael addition reaction, still some C=C groups can be observed in the spectrum.

Moreover, due to the hydrolysis of ester groups and the introduction of amino groups in 8PEG-NH_3, a new band around 3500 cm^{-1} can be observed.

2.3. Swelling Behaviour and Elasticity

Several experiments were conducted to get further insight into the swelling degree, the crosslinking density and corresponding mesh size, and the mechanical properties, i.e. stiffness, of gels prepared by the two different crosslinking chemistries. From the swelling ratio, as determined by the ratio of the swollen gel and the dried gel (see also Figure S4 below for the swelling/deswelling data), the average mesh size was calculated. The values are summarized in the following Table.

Table S1. Swelling ratio and mesh size as calculated for 8PEG-UV and 8PEG-NH_3 gels, respectively, and surface elasticity as measured by AFM (in air).

	Swelling ratio	Mesh size (nm)	Surface Elasticity (MPa)		
			dry	wet	drying
8PEG-UV	3.9	2.7	80	~1	30-40
8PEG-NH3	10	8.5	10 GPa	2.8	3-4 - < 1*

* 8PEG-NH3 gels disintegrate within several h in water, and also when drying after having been hydrated.

2.4. Melting and Crystallization Temperatures and Crystallinity

In the DSC melting and cooling cycles (Figure 3 in the manuscript), the typical melting and crystallization temperatures were recorded. Furthermore, the degrees of crystallinity were determined. The values are listed in Table S2.

Table S2. Melting and crystallization temperatures and degree of crystallinity as determined from the DSC analysis.

	Melting Temperature (°C)	Crystallization Temperature (°C)	Crystallinity (%)
8PEG	52	33	96
8PEG-NH3	51	29	82
8PEG-UV	38	22	67

3. AFM characterization of surface morphology

3.1. Dehydrated gels

Besides letting the as-prepared gels dry over the course of several hours in air (Figure 4 in the manuscript), they were put in a vacuum oven overnight at 40 °C to remove all water, including the hydration mantle from the PEG-gels. AFM studies were carried out to see if this would affect the morphology. Figure S3 demonstrates that, in this case, there was a rather regular texture observed at the dehydrated surface of the 8PEG-UV gels, whereas the surface of the dehydrated 8PEG-NH_3 was amorphous and irregular. Moreover, the 8PEG-UV surface exhibited a smooth topography, while there was a significant topographic landscape on the 8PEG-NH_3 gels.

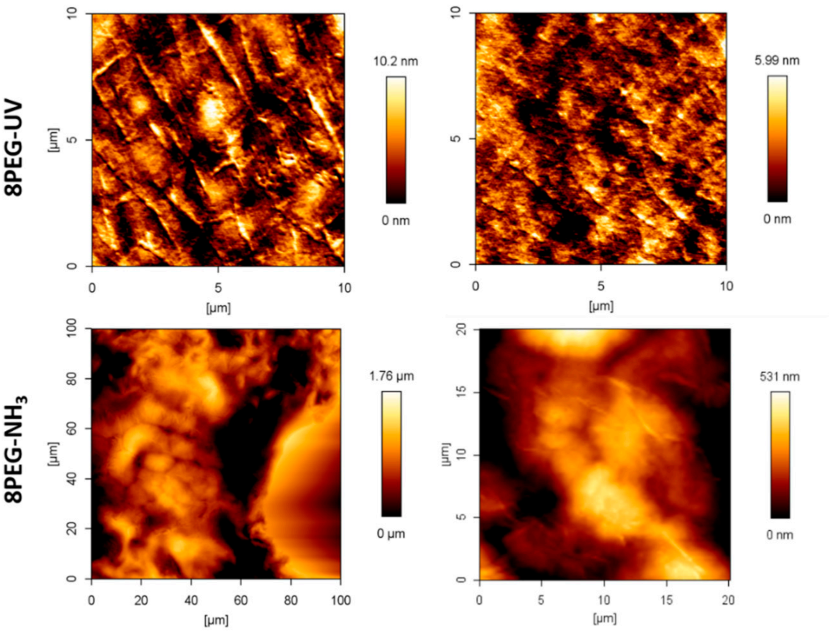

Figure S3 Representative AFM images of fully dehydrated 8PEG-UV (top) and 8PEG-NH_3 (bottom) gels. As-prepared samples had been dried overnight in a vacuum oven at 40°C prior to the measurements (in air).

3.2. Hydrated gels

The dehydrated gels were immersed in water until the equilibrium water content (EWC) was reached, and then analyzed by AFM, Figure S4, left. These gels with their EWC were left to dry again in air and analyzed by AFM during drying (measured in air), Figure S4 (middle and right):

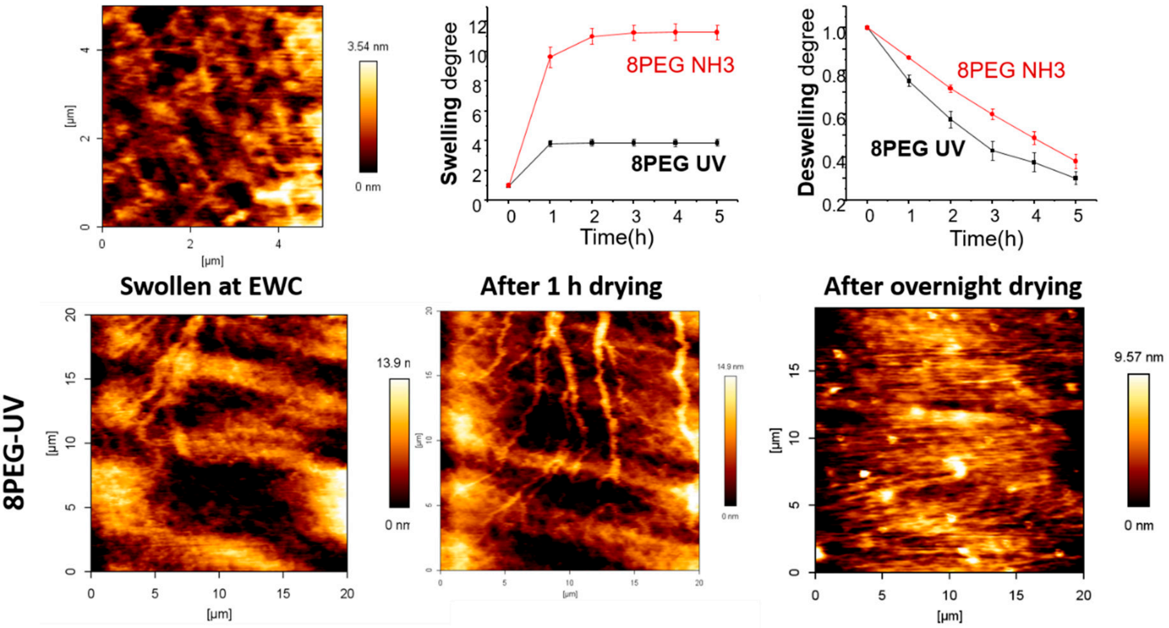

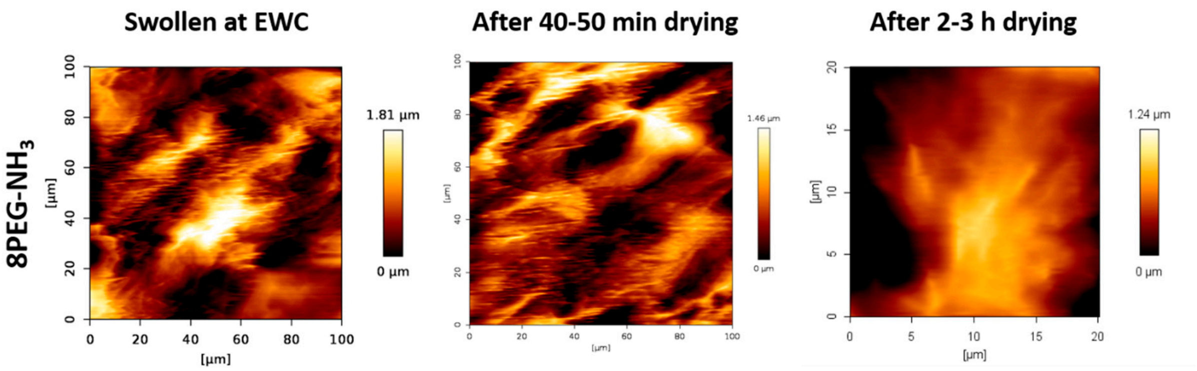

Figure S4 AFM height images of 8PEG-UV gels that were fully hydrated (left) and that were measured at different time points during drying. The graphs (inset) show the swelling and deswelling over time, for the two different hydrogels. .
